# Poultry Farmer Training in Biosecurity and Production Within an Evaluation Framework in Bangladesh

**DOI:** 10.1002/vms3.70773

**Published:** 2026-01-06

**Authors:** Meherjan Islam, Ayona Silva‐Fletcher, Easrat Jahan Esha, Syeda Munira Dilshad, Md. Ershadul Haque, Nurun Nahar Chisty, Rashed Mahmud, Md. Helal Uddin, Fiona Tomley, Md. Ahasanul Hoque

**Affiliations:** ^1^ Department of Medicine and Surgery Chattogram Veterinary and Animal Sciences University Chattogram Bangladesh; ^2^ Department of Clinical Science and Services Royal Veterinary College University of London Hatfield Hertfordshire UK; ^3^ Department of Pharmacy International Islamic University Chattogram Chattogram Bangladesh; ^4^ Department of Pathobiology and Population Science Royal Veterinary College University of London Hatfield Hertfordshire UK

**Keywords:** attitude, evaluation, knowledge, poultry farmers, practices, training

## Abstract

**Background:**

In Bangladesh, farmers often initiate small‐ to medium‐scale poultry production ventures with minimal to no prior training, experience or formal qualifications. The poultry industry's rapid expansion poses a growing threat to human and animal health. It is, therefore, imperative to educate farmers using the One Health approach, recognizing the interconnectedness of human, animal and environmental health.

**Objectives:**

This study aimed to train farmers with an evaluation framework to assess their knowledge, attitudes and practices regarding poultry biosecurity and management. The study also assessed the effectiveness of the training programme using the Kirkpatrick training evaluation model.

**Methods:**

The training intervention study was a series of 2‐day trainings with a survey before and after the training. Farmers were selected from small to medium‐scale broiler and Sonali farming sectors. The data were analysed using R‐software.

**Results:**

A total of 183 farmers participated in the training. They had good knowledge regarding correct brooding temperature, poultry vaccines, antibiotic names and whom to contact for advice on poultry‐related problems before training. The training was effective in improving knowledge gain for day‐old chick selection, causes of vaccine failure, the exact function of antibiotics, and antimicrobial resistance. A single training intervention is inadequate to change farmer behaviours, and continuous communication is therefore necessary.

**Conclusions:**

This study provides empirical evidence on the pre‐existing knowledge and the impact of training using different pedagogical approaches on biosecurity and the production of poultry farmers with diverse educational backgrounds and varied experience in farming—aligning Kirkpatrick model.

## Graphical abstract

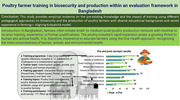

## 1 Introduction

The Bangladesh poultry sector is a rapidly growing subsector of agriculture. It employs eight million people directly or indirectly (Islam et al. 2021) with a total chicken population currently at 320 million, with around 90,000 registered commercial chicken farms (Ali and Chowdhury 2021; DLS 2023)

In recent years, animal protein consumption in Bangladesh has increased in line with the country's rising middle‐class population (ITA 2022), and poultry farming is a profit‐making commercial enterprise. In Bangladesh, two types of chickens are reared for meat: commercial exotic broilers (rearing period: 25–35 days) and Sonali (crossbred of male Rhode Island Red and female Fayoumi, rearing period: 60–65 days) (Islam et al. 2021)

Small‐ and medium‐scale commercial poultry farmers with 500–5000 birds per farm significantly contribute to fulfilling the demand for poultry meat and eggs (Shah et al. 2006; Begum et al. 2012). To maximize profit, they have to ensure the quality of day‐old chicks (DOCs), carefully manage their poultry feed, and effectively oversee all aspects of farm management (Yensuk 2019).

Small‐ and medium‐scale poultry (broiler and Sonali) farming in Bangladesh is feasible with minimal investment and technical knowledge due to support from dealers of private poultry feed and DOC‐production companies and poultry field veterinarians (Hennessey et al. 2021). However, it is a competitive business with an uncertain profit or even loss due to combinations of factors including disease outbreaks, inadequate management, poor‐quality DOCs, volatility in feed costs, changing farm‐gate prices, and the multiplicity of actors in the production and distribution networks, all of whom need to take a small profit for their survival (Hennessey et al. 2021; Kawsar et al. 2013; Huque et al. 2016).

Small and medium‐scale farmers may start with their own poultry sheds or, on a leased one (Kawsar et al. 2013). They commonly rear chickens in open‐sided sheds with wire or bamboo mesh, corrugated tin roofs and mud floors (Rimi et al. 2017). After receiving DOCs from dealers or poultry companies, they start by brooding broiler and Sonali chicks following the advice given to them by their dealers or local company veterinarians (Islam 2023). Throughout the rearing period, the dealers or company representatives provide feed obtained from the companies with whom they are directly or indirectly connected (Islam 2023).

Education is crucial for farmers to boost their knowledge and skills, and enable them to make informed decisions about their business (Begum et al. 2010). There are government‐organized training programs in Bangladesh for poultry farmers, for example, a 6‐month ‘Certificate in Livestock and Poultry’ training organized by Bangladesh Open University, a 3‐month ‘Youth Trained Center’ training scheme under the Ministry of Youth and Sport, and an ‘Upazila‐to‐Community Participatory Disease Searching’ program organized by the Department of Livestock Services (DLS) and FAO (Ershad et al. 2004; Brum et al. 2018; Orubu et al. 2020). However, the training offered by the government is limited due to insufficient manpower and budget (Islam 2023). Private companies, including feed and DOC production companies, along with pharmaceutical companies, often arrange local training sessions or workshops for poultry farmers (Islam 2023). These latter initiatives serve dual purposes; they allow companies to promote their products and enhance management practices. The impact of this type of training, which has a focus on marketing, has not been established (Islam 2023).

Poultry biosecurity is based on preventive measures designed to reduce the risk of transmission of infectious diseases, parasites and pests among poultry, between poultry and humans, and from the environment to poultry (Faroque et al. 2023; Alagesan et al. 2025). It involves practices that limit the introduction and spread of pathogens within and between poultry farms (Alagesan et al. 2025). One goal of existing farmer training programmes is to educate farmers on farm management and biosecurity measures, in addition to supporting them to be profitable and enabling them to adhere to standards assigned by both private and government poultry sectors (Islam 2023). These measures such as isolation of new and/or sick birds, traffic control to regulating the movement of people, animals, equipment and vehicles on and off the farm, sanitation to ensure regular cleaning and disinfection of facilities, equipment and footwear, health monitoring with regular health checks and vaccination programs, rodent and pest control to keep pests away as they can carry diseases, and controlled clean feed and water supply to prevent contamination should be a focus in farmer training to prevent potential future pandemics (Faroque et al. 2023; Alagesan et al. 2025; Amalraj et al. 2024).

In Bangladesh, the growth in small‐ and medium‐scale poultry enterprises is conducted within ‘fragile’ enterprises by inexperienced and poorly supported producers, many of whom lack capacity for the level of system upgrading needed to mitigate disease risks (Hennessey et al. 2021). Therefore, it is essential to educate farmers about zoonotic disease risks and the spread of antimicrobial‐resistant bacteria through the ecosystem to reduce any negative impacts that can occur on human health (WHO 2014).

Knowledge, attitude, behaviour and practice (KAP) surveys are widely used in non‐experimental social science research (Rav‐Marathe et al. 2016; Andrade et al. 2020). The KAP survey was chosen as the method of choice for this study due to its practical and context‐specific advantages. While other behavioural models—such as the Health Belief Model (Rosenstock 1974), Theory of Planned Behaviour (Ajzen 1991) and Social Cognitive Theory (Bandura 1999)—offer valuable psychological and social insights by addressing motivations, perceived barriers, social norms and behavioural intentions, they are often more suitable for designing complex or long‐term interventions. In contrast, the objective of the present study was to assess the existing knowledge, attitudes and practices (KAP) of poultry farmers and to evaluate whether a training program tailored to the Bangladeshi context could effectively influence these factors. Given this aim, a KAP survey provided a straightforward and effective approach for collecting relevant, actionable data.

To accurately gauge comprehension levels and skills development, it is imperative to conduct evaluations both before and after the training (Masta and Janjhua 2020). This facilitates understanding of the existing knowledge base among farmers with diverse educational backgrounds and farming experiences, and helps ascertain whether training objectives were successfully achieved (Masta and Janjhua 2020). The Kirkpatrick model of evaluation, originally developed in 1959 as a tool for assessing educational, training and learning programs, is suitable and applicable across various contexts (Alsalamah and Callinan 2022). In the Kirkpatrick model, outcomes are examined across four levels (Alsalamah and Callinan 2022). At the first level, the focus is on evaluating farmers' perceptions of the training program, providing valuable feedback to the training team. The second level assesses both the pre‐existing knowledge and what participants have actually learnt from the training intervention. The third level evaluates behavioural changes, determining how participants have applied their new knowledge in practice. Finally, the fourth level measures the overall impact of the training program on society (Alsalamah and Callinan 2022). The effectiveness of this evaluation model in farmer training (Diab 2015) and professional training (Kinnison et al. 2023) has been documented in previous research.

In training farmers from varied educational backgrounds, it is useful to select a diverse set of instructional strategies and teaching tools. These multiple educational methods tailored to different learning needs include an array of participatory and learner‐centred pedagogical approaches that include a combination of visual, verbal, experiential learning tools and problem‐based learning. Use of multimedia, for example, videos and visual resources, reduces cognitive load and enhances engagement (Mayer 2002; Tabbers et al. 2004). Another successful approach is problem‐based learning (PBL) and small group discussions (Dolmans and Schmidt 2006). In PBL, farmers can identify and discuss their own problems and learn from each other. Using real farms to conduct practical teaching and demonstrations is also useful, as farmers can be guided to identify strengths and areas to improve in their own farms.

The aim of this study was to train small‐ and medium‐scale broiler and Sonali farmers with an evaluation framework to assess existing KAP regarding poultry biosecurity and management, and to assess the effectiveness of the training programme using the Kirkpatrick training evaluation model. We hypothesized that a training programme designed with interactive approaches and modern pedagogical tools would change the KAP of farmers.

## 2 Materials and Methods

### 2.1 Study Design

**FIGURE 1:**
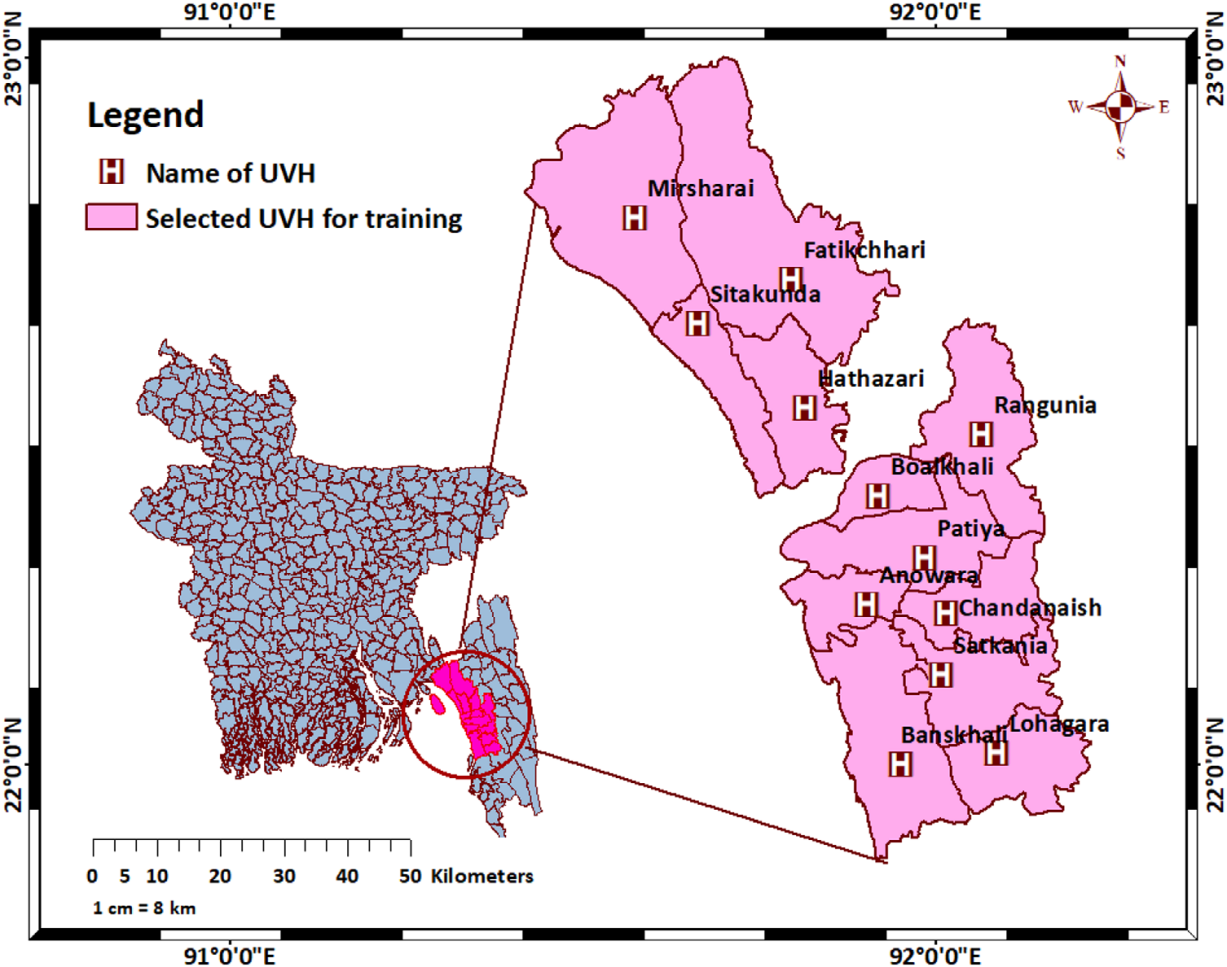
Distribution of the training areas (12 upazilas) of Chattogram district from July 2022–January 2023 (UVH: upazila veterinary hospital; H stands for hospital [i.e., veterinary hospital]).

A training intervention study with prospective data collection using pre‐ and post‐questionnaires was conducted with 183 farmers (based on logistical considerations and ensuring sufficient study power for meaningful study outcome) from 12 upazilas (sub‐districts) belonging to the Chattogram district (Figure 1). This district accommodated 18 million poultry distributed across 4882 broiler, 559 layer, 295 Sonali and 20 breeder farms (Foysal 2020). The methodological approach was grounded in community‐based research, incorporating the principles of Appreciative Inquiry—a participatory, strengths‐based approach aimed at fostering collective learning and sustainable community transformation (Whitney and Stavros 2008). This approach focused on identifying and building upon existing assets and capacities within the community, with the goal of enhancing and expanding the community's reserve capacity—its ability to respond effectively to challenges and opportunities (Whitney and Stavros 2008).

### 2.2 Participant Selection

Training programmes were held in 12 Upazila Livestock Office and Veterinary Hospital (ULO and VH), with 11–19 farmers attending, depending on the capacities of the training venue. Participants were selected using a non‐probability, convenience sampling method with the active support of ULO and VH. Though the target training population was for small—(< 500) and medium (up to 5000)—scale farmers, 33 farmers took part in the training, who were also included in this study. Farmers rearing chickens specifically for meat production rather than for egg laying were selected. The breeds selected are:

- Exotic Broiler, which refers to commercially bred chicken strains, such as Cobb 500 or Ross 308, developed outside the local region and known for fast growth, high feed efficiency, and large meat yield.
- Sonali, which is a locally popular crossbreed in Bangladesh, created by crossing Rhode Island Red (RIR) males with Fayoumi females. Sonali chickens are valued for their disease resistance, adaptability to local conditions, and dual‐purpose traits (meat and eggs), making them a favoured choice among small‐ and medium‐scale farmers.

The numbers for each training session were capped to enable effective small‐group teaching and interactive individual farmer participation.

### 2.3 Training Programme

The 2‐day training programme was developed with a One Health perspective, fostering collaboration among members of the Bangladesh One Health Poultry Hub (OHPH) team, the local ULO and VH, local medical doctors and private poultry feed companies. The approach used both classroom and field‐based activities, including interactive discussion sessions, PowerPoint, video and poster presentations, farm visits and question and answer sessions. The resource team included poultry experts and medical doctors, with additional online poultry expert participation.

The training included topics relevant to commercial poultry farming and One Health significance. The discussion sessions covered DOC quality, brooding management, vaccine application, transport, poultry farm biosecurity measures, common poultry diseases and their preventive measures, antimicrobial usage and management of market‐ready chickens in the final week of rearing. A poster presentation was used to illustrate government policies regarding poultry farm registration, antimicrobial usage (AMU), the Animal Disease Act‐2005, Animal Feed Act‐2010 and Animal Welfare Act‐2019 (UNIDO 2023). There were sessions with a medical doctor to discuss One Health approaches using examples of AMU and risk of zoonosis, including the role that farmers can play in preserving the One Health concept. Group tasks on chicken farms taught farmers how to monitor and maintain biosecurity using established examples from the farm and with assistance from poultry experts, both on‐site and online, to address their questions and concerns.

### 2.4 Pre‐ and Post‐Training Data Collection

A structured questionnaire was developed with 14 questions (Supporting Information Appendix 3) related to poultry farming KAP, including common poultry diseases, vaccination, biosecurity measures and AMU. The core research team was involved in preparing, reviewing, and finalizing the questionnaire, which was piloted by the lead author and revised based on the responses. The questionnaire was prepared in both Bengali and English, and the Bengali version was delivered to the farmers. Farmers completed the questionnaire as a pre‐training survey immediately before the training, which helped them to realize what they were going to learn from that training and helped them to assess their learning needs from the training. At the end of the 2‐day programme, the same questionnaire was repeated as a post‐training survey. A coding system was used to identify the pre‐ and post‐questionnaires of each farmer. A large number of farmers completed the questionnaire themselves, but some needed support in writing their responses, and the training team assisted them without introducing biases. Farmer consent was obtained before conducting the training and surveys.

### 2.5 Data Analysis

Data from the questionnaires were transferred manually by researchers to MS 2021 for storage. Data sets were cleaned, sorted and coded to generate the required processed data sets. After that, data sets were exported to R‐software version 4.2.2. (a programming software supported by the R Foundation for Statistical Computing, created at the University of Auckland and developed by the R Development Core Team) for analysis.

Depending on whether there were categorical (DOCs quality, brooding temperature, disease recognition, whom to take veterinary consultancy, vaccination practices and vaccine failure causes, selecting biosecurity practices from the given list, antibiotic function, selecting only antibiotics from the given list) or binary responses (Heard the word “Biosecurity”, knew about AMR), either chi‐test or Fisher's exact test was performed to assess the statistical significance of the responses of each question between pre‐ and post‐survey.

## 3 Results

### 3.1 Distribution of Participating Farmers for the Training

The number of farmers who participated in the training program from different upazilas in Chattogram district ranged from 11 to 19 (Table 1).

**TABLE 1:** Distribution of participating farmers in different upazilas of Chattogram district.

Name of upazilas	Number of broiler farmers (*n* = 140)	Number of Sonali farmers (*n* = 30)	Number of farmers with broiler and Sonali (*n* = 13)	Total number of farmers (*n* = 183)
Anowara	7	5	3	15
Banshkhali	11	4	0	15
Boalkhali	14	3	2	19
Chandanaish	12	4	1	17
Fatikchhari	12	4	0	16
Hathazari	10	2	3	15
Lohagara	10	2	0	12
Mirsharai	18	0	0	18
Patiya	10	1	0	11
Rangunia	12	2	0	14
Satkania	11	2	2	15
Sitakunda	13	1	2	16

### 3.2 Characteristics of Farmers and Farms

Among participating farmers (183), 95% were male. Their education levels were: no formal education (7%), primary (16%), secondary (32%), higher secondary (31%), graduate (9%) and post‐graduate (5%). Poultry farming was the main income source of most farmers (74%) with an experience of < 2 years (32%), 2–7 years of poultry farming (35%), > 7 to 13 years (21%) and > 13 years (12%). All participants operated small to medium‐scale farms (< 500–5000 chickens) and were broiler farmers (77%), Sonali farmers (16%), or both broiler and Sonali farmers (7%). There were 4 types of investment categories across the farms, which operated on partial credit (32%), full credit (31%), cash (21%) or contract (16%). Most farms had two sheds to rear poultry (37%), followed by one shed (32%), three sheds (16%) and four or more sheds (15%). Only 20% of the farmers registered their poultry farms with the government between 2008 and 2023 (Supporting Information Appendix 1).

### 3.3 Farmer's Knowledge, Attitudes and Perceptions of Various Aspects of Farm Management

#### 3.3.1 Status of Farmers’ Knowledge of Poultry Farm Management

**FIGURE 2:**
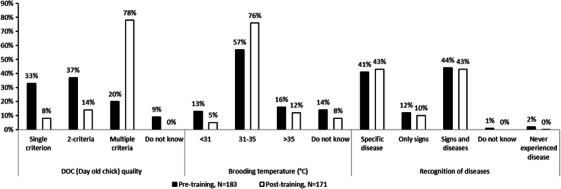
Status of farmers’ knowledge of poultry farm management. DOC quality: weight, active, colour, eyes, beak, feather, legs, navel, vent, source, strain, previous performance. Disease: Newcastle disease, infectious bursal disease, colibacillosis, salmonellosis, brooder pneumonia, coccidiosis, fowl cholera, fowl pox, complex respiratory disease, avian influenza, Marek's disease, necrotic enteritis, infectious coryza, fungal disease. Signs: depression, nasal discharge, fever, coughing, ascites, lameness, unabsorbed yolk sac, bloody diarrhoea, white faeces, brown faeces, kidney damage, shrunken legs, inappetite, dyspnoea, swollen eyes, heat stroke, gout.

In general, farmers’ knowledge of assessing DOC quality criteria before the training was low, with the largest proportion (37%) identifying only two criteria of DOC quality before the training. After training, 78% of farmers were capable of identifying multiple criteria for pre‐purchase checking of DOC quality, which was a highly significant increase (*p* < 0.001) (Figure 2). Knowledge regarding correct brooding temperature was 57% before training and this increased to 76% after the training (*p* = 0.013) (Figure 2) however, disease recognition knowledge, both regarding specific diseases and signs of diseases, did not show any improvement (41% in pre‐training and 43% in post‐training, *p* = 0.7) (Figure 2).

#### 3.3.2 Support of Poultry Veterinary Health Care Services

**FIGURE 3:**
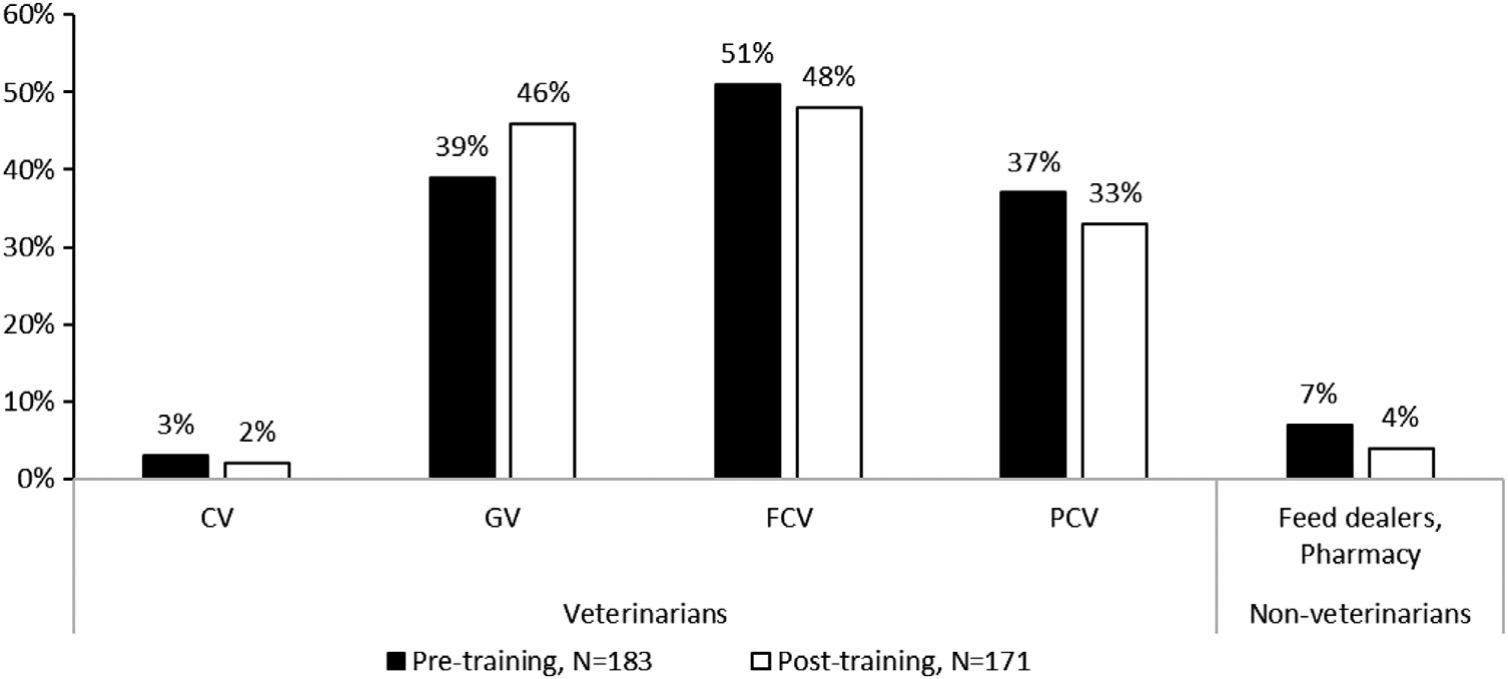
Whom to communicate with for poultry veterinary health services (note that most of the cases farmers gave multiple responses). CV, consultant veterinarian; GV, government veterinarian; FCV, feed company veterinarian; PCV, pharmaceutical company veterinarian.

A very high proportion of the farmers knew that they should contact a veterinary professional to get advice. Farmers’ perceptions of how they might get professional advice and who to contact in the first instance showed only minor changes, with 93% opting to contact a veterinary professional before training and 96% after training (*p* = 0.3). The 7% of farmers who contact non‐veterinarians for advice showed a minor decline after the training to 4% (*p* = 0.3) (Figure 3). A minority of the new‐starter farmers (1%) did not take consultancy from anyone, as there was no need for advice due to the lack of disease in their farm.

#### 3.3.3 Farmer's Knowledge of Vaccination, Practices and Causes of Vaccine Failure

**FIGURE 4:**
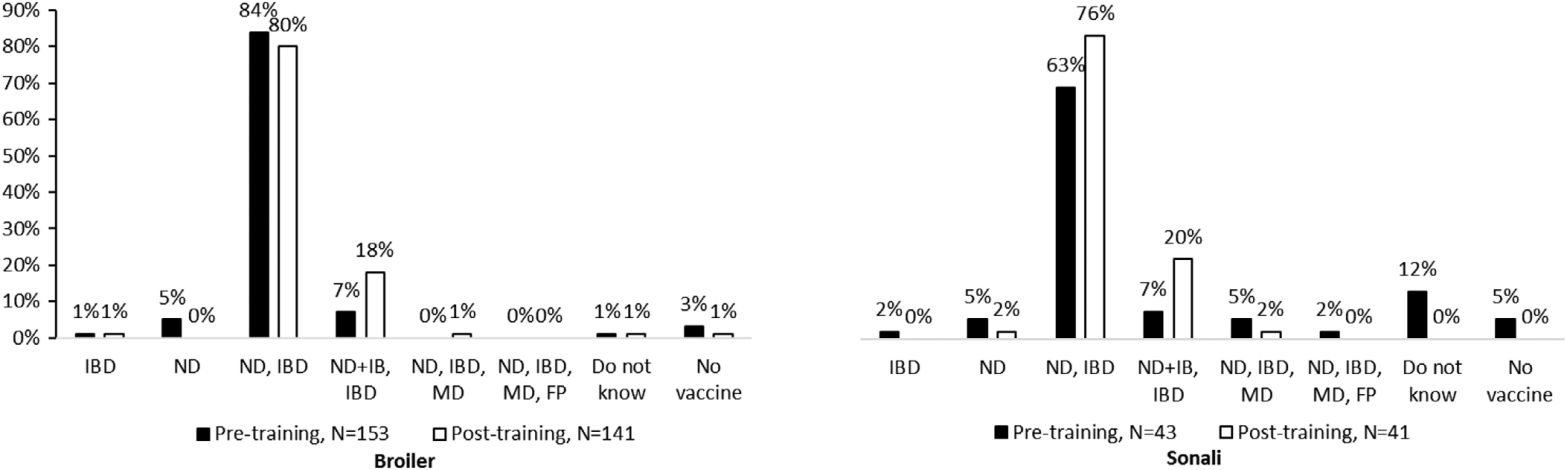
Knowledge of vaccination in poultry farms in different upazilas of Chattogram, Bangladesh (pre‐training: 153 broiler and 43 Sonali farmers; post‐training: 141 broiler and 41 Sonali farmers, the farmers who reared both broiler and Sonali chickens were counted separately). IBD, infectious bursal disease; ND, Newcastle disease; ND+IB, combined vaccines with Newcastle disease vaccine and Infectious bronchitis vaccine; MD, Marek's disease; FP, fowl pox.

Both Broiler and Sonali farmers had good pre‐training knowledge regarding vaccination practices for Infectious bursal disease (IBD) and Newcastle disease (ND), broiler (84%) and Sonali farmers (63%), respectively (Figure 4). The only noticeable increase in knowledge in post‐training was seen for dual combined vaccines (combined ND and Infectious bronchitis vaccine), which increased to 18% for broiler farmers (from 7%, *p* = 0.011) and to 20% for Sonali farmers (from 7%, *p* = 0.1) (Figure 4).

The farmer's knowledge of why diseases continue to occur after vaccination showed a marginal improvement after training. The training intervention caused a reduction in the percentage of farmers who did not know why diseases continued after training decreased from 38% to 22% (*p* < 0.001). About 3% of the farmers expected the vaccines to kill the infection‐causing organisms, and this reduced to 2% (*p* = 0.3) after training.

Improper management was cited more frequently after training as a reason for disease occurrence in the chickens after vaccination, from 16% to 20% (*p* = 0.081). Moreover, an increased percentage of farmers learned the specific causes (from 48% to 67%, *p* < 0.001) of disease occurrence after training, for example.

**FIGURE 5:**
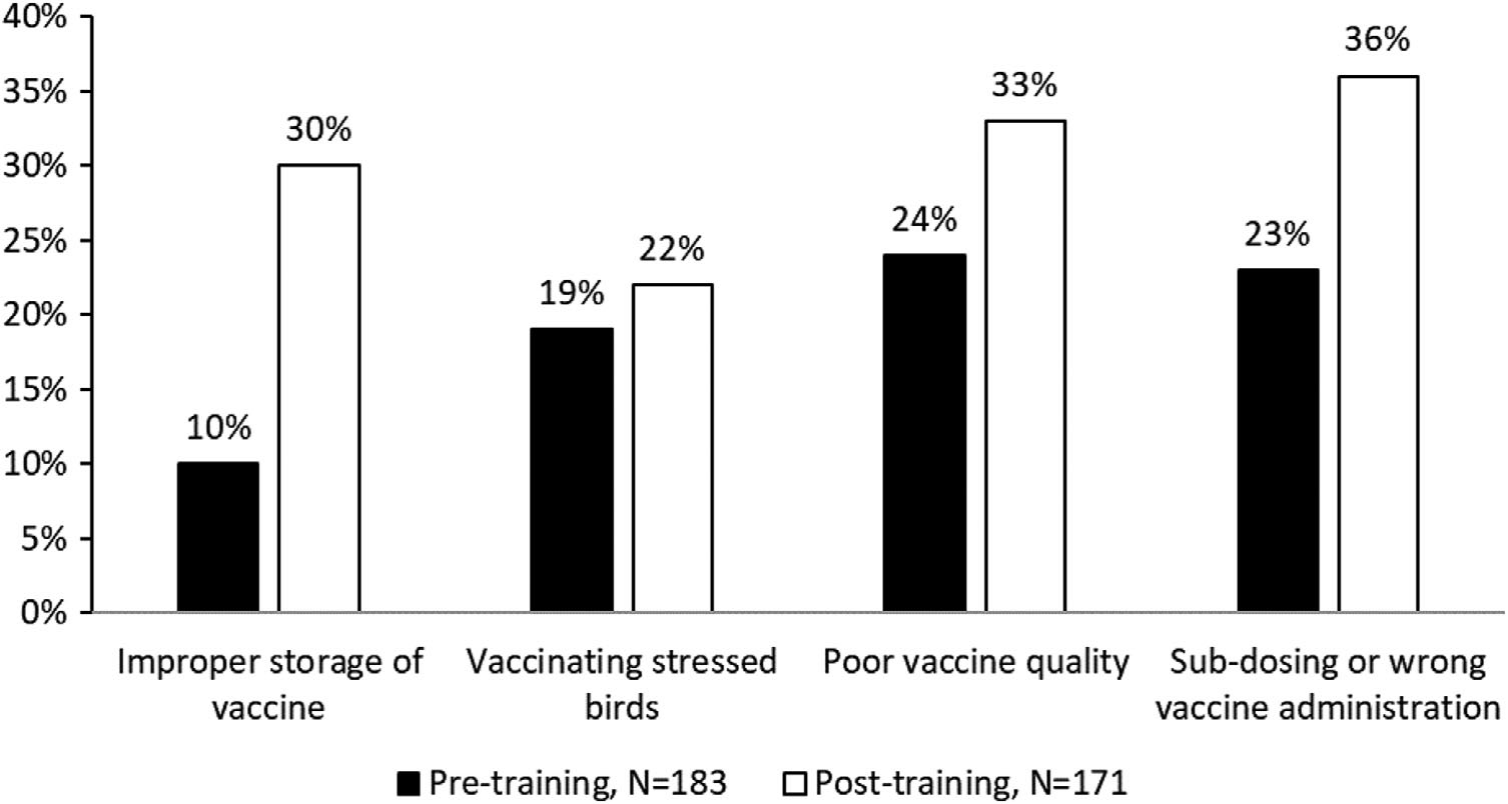
Farmers’ perceptions about some specific causes of disease occurrence after vaccination; single to multiple responses by each farmer and responses were counted individually.

Vaccine failure was attributed to improper vaccine storage, and this knowledge increased from 10% to 30% with training (*p* < 0.001). Knowledge regarding vaccinating stressed chickens (from 9% to 22%, *p* = 0.9) and poor vaccine quality (from 4% to 33%, *p* = 0.9) did not change with the training. Farmers gained knowledge that sub‐dosing or wrong vaccine administration was a reason for vaccine failure due to training (from 3% to 36%, *p* = 0.03) (Figure 5). Before training, 4% of the participants expected vaccines could not kill organisms, which decreased to 0% after training (*p* = 0.2).

#### 3.3.4 Farmer's Knowledge of Biosecurity, Antibiotics and Antimicrobial Resistance

##### 3.3.4.1 Level of Farmers’ Knowledge of Biosecurity Measures

**FIGURE 6:**
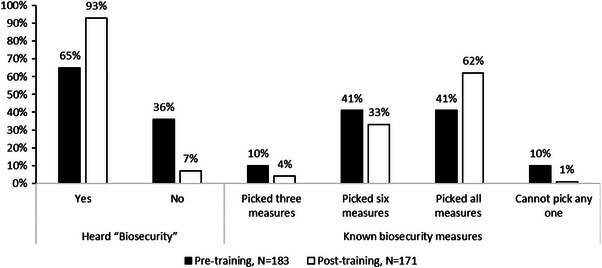
Farmers’ knowledge of biosecurity measures at different aspects. Given biosecurity measures: Entrance restriction for visitors or outsiders, Protecting birds from thief at night, Restriction in vehicle movement, Protecting fence surrounding the farm, Using rat trap, clean water supply, ventilation and waste management, Cleaning and disinfecting inside and outside of the farm regularly, Discarding dead and sick birds properly, Personal hygiene maintain to the farm labours.

Overall, 41% of farmers had a good knowledge regarding biosecurity measures and were able to pick all the biosecurity measures correctly from the given list in the pre‐survey (Figure 6). After training, this increased to 62% (*p* < 0.001). Interestingly, farmers’ knowledge at the outset of the word ‘biosecurity’ was lower than their actual knowledge of specific practices that contributed to good biosecurity. After the training, most farmers (93%, *p* < 0.001) knew the word ‘biosecurity’. The most significant result of the training was a change in the proportion of farmers who did not know any biosecurity measures from 10% to 1% (*p* < 0.001) (Figure 6).

##### 3.3.4.2 Level of Farmers’ Knowledge of Antibiotics and Antibiotic Resistance

**FIGURE 7:**
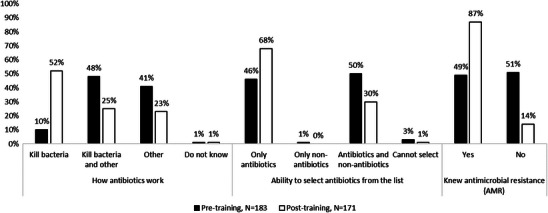
Farmer's knowledge regarding antibiotics and antimicrobial resistance at different aspects. Other: kill virus, prevent diseases, increase body weight and increase immunity. Antibiotics: amoxicillin, doxycycline, oxytetracycline, ciprofloxacin, tylosin, neomycin, colistin, sulfaclozine. non‐antibiotics: toxin binder, immuno‐modulator, renal tonic, multi‐vitamin, paracetamol, fevasol, probiotics.

In the pre‐survey, a total of 46% of farmers were able to identify antibiotics from the given medicines list, though only 10% knew the function of antibiotics. After training, an increased number (52%, *p* < 0.001) learned the function of antibiotics (68%, *p* < 0.001) selected only antibiotics from the same given list (Figure 7). These results also showed that farmers' awareness of AMR increased almost twofold (87%, *p* < 0.001) after training (Figure 7).

#### 3.3.5 Farmers’ Expectations and Learned Topics From the Training

Table 2 summarizes the answers farmers gave about what they expected to learn before the training and what they felt they had learned afterward. A considerable proportion of the farmers wanted to learn about poultry farm management (58%), poultry diseases and disease preventive measures (44%), and antibiotics and vaccine application (32%), as evidenced by the pre‐training survey. The post‐training survey showed that farmers felt they had learned about farm biosecurity measures (48%), antimicrobial usage and AMR (47%), poultry vaccination (39%), how to make good quality DOC selection (27%), farm management (26%) and brooding management (23%).

**TABLE 2:** Farmer expectation and assessment of learning (multiple responses are counted individually).

Expected to learn (*N* = 183 pre‐training)	%	Learned topics (*N* = 171 post‐training)	%
Nothing	5	Nothing	5
Do not know what to ask	1	Learned but did not mention the topic	13
Different diseases and the preventive measures	44	Disease management	15
Specific Infectious diseases (IBD/ND/Coli/Coccidiosis/BP)	14	Importance of vet consultancy	4
Non‐infectious diseases (ascites, stroke)	4	Specific laws regarding the poultry sector	10
Disease conditions (nasal discharge, lameness, mortality)	7	Quality of a good poultry farmer	1
Medicines (antibiotics, vaccines)	32	Antimicrobial usage and AMR	47
		Vaccination protocols	39
Farm management (shed preparation, day‐old chick selection, brooding, feeding management, biosecurity measures, modern technology of poultry farming, communication with the vet)	58	Shed preparation	1
		Use of curtain to ensure farm ventilation	4
		Brooding management	23
		Feed management	2
		Biosecurity management	48
		Litter management	10
		Good quality chick identification	27
		Farm management	26
Ready birds’ profitable marketing	3		
Different species rearing (layer farming, guinea fowl rearing, cattle farming, organic chicken rearing)	3		
Government initiatives (Training provision for the farmers, easy loans, monitoring feed, chick and ready birds price market)	7		

#### 3.3.6 Influence of Farmers’ Experience in Gaining Knowledge From the Training

All the farmers showed a significant increase in their knowledge of DOC quality, causes of vaccine failure, select biosecurity measures from a given list, knowledge of antibiotic function, select antibiotics from a given list, or whether they knew of AMR. The experience of farmers (less than or more than 4 years) had no effect on farmers increase in knowledge on any of the above (Supporting Information Appendix 2).

## 4 Discussion

The farmers who participated in the study had a range of experience and were highly dependent on poultry farming for their livelihood. Therefore, a reasonable knowledge of practices related to poultry production and biosecurity measures was to be expected at the outset and was evidenced by the study results. The results of this 12‐site programme demonstrated that training can improve farmers’ knowledge across many aspects of poultry farming, for example, improving knowledge regarding DOCs selection. However, knowledge gain in some topics, for example, disease recognition and disease signs, was low.

A variety of pedagogical tools and approaches were employed to ensure the training programme met the needs of all the farmers. To maximize farmer engagement, an interactive approach was adopted between farmers and the expert trainers. The experts, who were reputable poultry practitioners, were highly regarded by the farmers in the region. The training covered ‘DOC selection criteria and process’ using ‘Pasgar's scoring technique’ (Narinç and Aydemir 2021) for the identification of high‐quality chicks. Farmers were particularly engaged with this topic as they recognized the value of this technique to negotiate with their associated dealers for better quality chicks and to justify replacements when poor quality chicks were supplied. They also learned the importance of separating weak or underweight chicks and caring for them separately. Regarding brooding temperature, some farmers had a wrong conception of providing 1 W of power (26°C) per bird was sufficient. Through discussion and expert opinions, most farmers learned the importance of measuring the shed's temperature before determining the necessary wattage, as excessive heat could be harmful.

Farmers primarily focus on maintaining the economic balance of their poultry enterprise. When their flocks fall ill, they typically seek medication from their dealers rather than implementing preventive biosecurity measures. Some farmers do consult veterinarians, but their numbers are limited. The training emphasized the importance of consulting registered veterinarians before obtaining prescriptions and medications, which helps to reduce treatment costs, ensuring only essential medicines are used.

As shown in Figure 3, farmers were aware of veterinarians before training, but mostly relied on private company veterinarians for advice and acquiring feed and DOCs. When these private company veterinarians visited their farms, the farmers would seek their advice on medications and strategies for growing their birds to optimum selling weight. Despite this, they took the medicines, particularly antibiotics, from the local veterinary pharmacies or dealer shops after discussions with the dealers. After the training, farmers changed their perspective, recognizing the importance of consulting veterinarians for veterinary advice.

The training addressed farmers’ perceptions of vaccines, how they work and why they sometimes fail, revealing that (84% of the participants) farmers already had excellent knowledge and practices regarding vaccines. When starting a broiler or Sonali flock, farmers commonly obtain a vaccination schedule from the veterinarians, the neighbouring farmers or dealers (Islam 2023). However, they rarely recheck or update this schedule. Sometimes, they miss scheduled vaccines due to financial constraints or neglect the sickness of their chickens rather than consulting a veterinarian. The training delivered a clear message about the importance of careful vaccination practices. Farmers gained crucial knowledge about why vaccines fail and how to prevent such failures. Prior to the training, farmers often blamed vaccine failure on factors such as poor or expired vaccines, cross‐matched or low‐quality vaccines. After the training, farmers understood the importance of maintaining vaccine quality, for example improving vaccine transport conditions, recognizing that they should focus on aspects they can control instead of solely blaming the vaccine quality.

Despite their prior knowledge of certain disease prevention measures, the farmers neglected to implement them because they believed that maintaining preventive measures would be more expensive and strenuous. For this reason, farmers paid less attention to biosecurity measures. However, after training, they realized that following biosecurity measures would help prevent diseases and reduce medicine costs.

The training effectively educated farmers on the antibiotic functions. Each session included a medical doctor alongside the poultry experts to help farmers understand their significant role by providing an affordable protein source to society. The experts emphasized the importance of reducing the irrational use of antibiotics, highlighting how farmers could contribute to reducing AMR.

The trainers explained the growing issue of AMR, showing how overuse of antibiotics in poultry affects both animal and human health. Given that farmers are often profit‐oriented, they were more receptive when they realized that improper antibiotic use could lead to farm and family losses (Sharma et al. 2022). Consequently, when informed about AMR and its implications, they prioritized finding alternatives, for example supplements and improved management practices, as suggested by the experts.

The training team evaluated pre‐surveys after the first day's session, and used the second day to address farmers’ mistakes and impart accurate knowledge. This helped the farmers to build confidence, as evidenced by the post‐survey results. The involvement of medical professionals in the training programme underscored the One‐Health concept, reinforcing the farmers’ sense of their societal importance and potential contributions. The presence of knowledgeable veterinary and medical professionals significantly impacted the farmers' attitudes and reactions (Ahmed et al. 2025).

The training was structured using an evaluation method that incorporated various teaching techniques, as discussed earlier. According to the Kirkpatrick model, this study focused on the first two phases of an evaluation‐based training program: the reaction phase and the learning phase. The study detailed how the training was delivered and highlighted the enthusiastic engagement of the farmers during the sessions. The results from the pre‐ and post‐surveys demonstrated the knowledge gained by the farmers, aligning with the second phase of the Kirkpatrick model.

Expecting poultry farmers to fully adopt new knowledge and practices after just one training program is challenging. It is crucial to establish ongoing communication and support systems to facilitate continuous learning and implementation. Many farmers are proficient in utilizing social media for communication, suggesting a potential avenue for future engagement.

Currently, a follow‐up study is in progress to examine the long‐term effects of the training and to assess any changes in behaviours and practices over time. As the farmers were recruited conveniently, this might limit the generalizability of the findings.

## 5 Conclusion

Training encompasses the process of educating, learning, and developing individuals to enhance their job performance by facilitating targeted knowledge transfer and skill enhancement. By utilizing improved pedagogical approaches and tools, the training programme successfully enhanced the knowledge gain of farmers with varying educational backgrounds.

The pre‐ and post‐surveys were a valuable approach in assessing the existing knowledge of farmers, which had not been documented before. However, some areas of farmers’ interests could not be covered in this training programme, which were found in the pre‐survey. The feedback provides useful information for designing future programmes, allowing prioritization of topics that align with the farmers' expectations and requirements.

## Author Contributions

**Meherjan Islam**: conceptualization, methodology, software, formal analysis, data curation, investigation, writing – original draft, writing – review and editing, visualization, validation. **Ayona Silva‐fletcher**: conceptualization, funding acquisition, writing – review and editing, project administration, supervision, resources, validation. **Easrat Jahan Esha**: data curation, writing – review and editing. **Syeda Munira Dilshad**: data curation, writing – review and editing. **Md. Ershadul Haque**: data curation, writing – review and editing. **Nurun Nahar Chisty**: data curation, methodology, writing – review and editing. **Rashed Mahmud**: data curation, writing – review and editing. **Helal Uddin**: data curation, writing – review and editing. **Fiona Tomley**: supervision, resources, project administration, conceptualization, funding acquisition, writing – review and editing, validation. **Md. Ahasanul Hoque**: conceptualization, investigation, funding acquisition, writing – review and editing, visualization, methodology, formal analysis, project administration, supervision, resources.

## Funding

This project is funded by a grant from the UK Research and Innovation Global Challenges Research Fund One Health Poultry Hub (BB/S011269/1), one of 12 interdisciplinary research hubs funded under the UK government's Global Challenges Research Fund Interdisciplinary Research Hub initiative.

## Ethics Statement

The Ethical Approval has been collected from Chattogram Veterinary and Animal Sciences University, Bangladesh, Ethics application no CVASU/Dir (R&E) EC/2020/165/2/1.

## Conflicts of Interest

The authors declare no conflicts of interest.

## Supporting information




**Supporting Appendix 1**: Demographics of training participants and their associated farms.

**Supporting Table**: Assess the influence of farmers’ experience on improving farmers’ KAP through the training program.

**Supporting File 3**: vms370773‐sup‐0003‐appendix3.docx.

## Data Availability

The data that support the findings of this study are available from the corresponding author upon reasonable request.
