# Supplementary material for: Poultry Farmer Training in Biosecurity and Production Within an Evaluation Framework in Bangladesh
Source: Vet Med Sci. 2026 Jan 6;12(1):e70773. doi: 10.1002/vms3.70773 (PMC12774789; doi:10.1002/vms3.70773)
Supplement: Supplementary file 1 — Supporting Appendix 1: Demographics of training participants and their associated farms. [file VMS3-12-e70773-s003.docx]

Appendix 1: Demographics of training participants and their associated farms

| Characters | Categories | n (%) |
| --- | --- | --- |
| Gender | Male | 173 (94.5) |
|  | Female | 10 (5.5) |
| Education | No formal education | 12 (6.6) |
|  | Primary | 29 (15.9) |
|  | Secondary | 58 (31.7) |
|  | Higher Secondary | 57 (31.2) |
|  | Graduate | 17 (9.3) |
|  | Postgraduate | 10 (5.5) |
| Farming experience (Year) | 0-2 | 58 (31.7) |
|  | >2-7 | 64 (35) |
|  | >7-13 | 38 (20.8) |
|  | >13-40 | 23 (12.6) |
| Income source | Poultry farming | 135 (73.8) |
|  | Poultry farming and any other source* | 30 (16.4) |
|  | Poultry farming and other two sources* | 5 (2.7) |
|  | Other sources | 13 (7.1) |
| Production type | Broiler | 140 (76.5) |
|  | Sonali | 30 (16.4) |
|  | Both (Broiler and Sonali) | 13 (7.1) |
| Investment (Financed) | Cash | 39 (21.3) |
|  | Contract | 29 (15.9) |
|  | Credit | 57 (31.2) |
|  | Partial credit | 58 (31.7) |
| Number of sheds | 1 | 59 (32.2) |
|  | 2 | 68 (37.2) |
|  | 3 | 29 (15.9) |
|  | 4 or more | 27 (14.8) |
| Farm registered | Yes | 37 (20.2) |
|  | No | 146 (79.8) |
| Flock size (broiler farms) | Small (<500-2500) | 106 (58) |
|  | Medium (2501-5000) | 52 (28) |
|  | Large (>5001) | 25 (14) |
| Flock size (Sonali farms) | Small (<500-2500) | 28(65.1) |
|  | Medium (2501-5000) | 7(16.3) |
|  | Large (>5001) | 8(18.6) |

[*Other sources: Agricultural shop, Sale center, different poultry farming, cattle farming, cultivation, aquaculture, rice mill, sewing, driving, computer and photocopy shop, house rent, pharmacy, business, NGO, service, income of the male members of the family]
