# Supplementary material for: Poultry Farmer Training in Biosecurity and Production Within an Evaluation Framework in Bangladesh
Source: Vet Med Sci. 2026 Jan 6;12(1):e70773. doi: 10.1002/vms3.70773 (PMC12774789; doi:10.1002/vms3.70773)
Supplement: Supplementary file 2 — Supporting Table: Assess the influence of farmers’ experience on improving farmers’ KAP through the training program. [file VMS3-12-e70773-s001.docx]

**Table**: Assess the influence of farmers’ experience on improving farmers’ KAP through the training program

| **Criteria** | **Sub-categories** | **Experience** $\boldsymbol{\leq}$**4years** | | | **Experience >4years** | | |  |
| --- | --- | --- | --- | --- | --- | --- | --- | --- |
|  |  | **Pre-survey (N=87)**  **% Responses** | **Post-survey (N=82)**  **% Responses** | **p** | **Pre-survey (N=96)**  **% Responses** | **Post-survey (N=89)**  **% Responses** | **p** | **Post-survey:**  $\boldsymbol{\leq}$**4yr % vs. >4yr %; p** |
| **DOC quality** | None/Single | 51 | 6 | <0.001 | 35 | 10 | <0.001 |  |
|  | Double or more | 49 | 94 |  | 65 | 90 |  | 94 vs. 90; 0.3 |
| **Vet communication** | Veterinarian | 91 | 96 | 0.14 | 93 | 93 | 0.88 |  |
|  | Non-veterinarian | 9 | 4 |  | 7 | 7 |  |  |
| **Causes of vaccine failure** | Specific cause | 32 | 51 | 0.03 | 35 | 53 | 0.027 | 51 vs. 53; 0.8 |
|  | Specific cause and other* | 12 | 15 |  | 19 | 16 |  |  |
|  | Other* | 16 | 8 |  | 12 | 13 |  |  |
|  | Do not know | 40 | 26 |  | 34 | 18 |  |  |
| **Select biosecurity measures from the given list** | 0-3 | 18 | 4 | 0.002 | 18 | 6 | 0.0012 |  |
|  | 4-6 | 42 | 35 |  | 43 | 31 |  |  |
|  | 7-9 | 40 | 61 |  | 39 | 63 |  | 61 vs. 63; 0.8 |
| **Knowledge of antibiotic function** | Do not know | 1 | 0 | <0.001 | 0 | 1 | <0.001 |  |
|  | Kill bacteria | 6 | 55 |  | 15 | 49 |  | 55 vs. 49; 0.4 |
|  | Kill bacteria and other | 45 | 21 |  | 51 | 29 |  |  |
|  | Other | 48 | 24 |  | 34 | 21 |  |  |
| **Select antibiotics from the given list** | Antibiotic | 40 | 71 | <0.001 | 51 | 66 | <0.001 | 71 vs. 66; 0.4 |
|  | Antibiotics and non-antibiotics | 52 | 27 |  | 48 | 34 |  |  |
|  | Do not know | 7 | 2 |  | 0 | 0 |  |  |
|  | Non-antibiotics | 1 | 0 |  | 1 | 0 |  |  |
| **Knew AMR** | Yes | 40 | 87 | <0.001 | 56 | 87 | <0.001 | 87 vs. 87; 1.0 |
|  | No | 60 | 13 |  | 44 | 13 |  |  |

[Other*= Non-specific causes, not make sense type causes, no vaccination, never experienced disease after vaccination]
