# Supplementary material for: Poultry Farmer Training in Biosecurity and Production Within an Evaluation Framework in Bangladesh
Source: Vet Med Sci. 2026 Jan 6;12(1):e70773. doi: 10.1002/vms3.70773 (PMC12774789; doi:10.1002/vms3.70773)
Supplement: Supplementary file 3 — Supporting File 3: vms370773‐sup‐0003‐appendix3.docx. [file VMS3-12-e70773-s002.docx]

Evaluation of knowledge, attitude and practices of small to medium-scale commercial broiler and Sonali farms located in Chattogram district, Bangladesh (Same questionnaire for pre- and post-survey)

Name of farmer: Gender: Male/Female

Address

Education: a) No formal education, b) Primary, c) SSC, d) HSC, e) Bachelor, f) Master’s

Mobile number: Farming experience: Main profession: a) Poultry farming, b) Other

1. Farm type: a) Broiler, b) Sonali

2. Number of birds: 3. Number of sheds:

5. Economic investment: a) Credit, খ) Half-credit গ) Cash ঘ) Contracted

6. Is the farm registered: a) Yes, year: ___________ b) No

7. What is the quality of a good day-old chick?

8. What is the ideal brooding temperature of day-old chick?

9. Which diseases do you mostly find in your farm?

10. What are the necessary vaccines for broiler and Sonali?

11. Why birds get diseased even after vaccination?

12. Have you heard the term “Biosecurity” before? a) Yes, b) No

13. Pick the biosecurity measures from the given list below:

a) Entrance restriction for visitors or outsiders, b) Protecting birds from thief at night, c) Restriction in vehicle movement, d)

Protecting fence surrounding the farm, e) Using rat trap, clean water supply, ventilation and wastage management, f) Cleaning

and disinfecting inside and outside of the farm regularly, g) Discarding dead and sick birds properly, h) Personal hygiene maintain

to the farm labours

14. What is your perception regarding antibiotic’s work?

a) disease prevention, b) kill bacteria, c) kill virus, d) increase body weight, e) increase body immunity, f) others

15. Select antibiotics from the given list:

a) Toxin binder, b) Immuno-modulator, c) Amoxicillin, d) Doxycycline e) Renal tonic, f) Oxytetracycline, g) Ciprofloxacin, h) Tylosin,

i) Multi-vitamin, j) Paracetamol, k) Neomycin, l) Colistin, m) Fevasol, n) Probiotics, o) Sulfaclozine

16. Do you know about antimicrobial resistance? a) Yes, b) No

17. Do you stop using medicines/antibiotics before selling your chickens? a) Yes, b) No, if yes, days……………………

18. Do you take veterinarian’s consultancy? a) Yes, b) No

19. Do you think that good farm management will benefit your farm economically?

20. What is your expectation (pre-survey)? Are you satisfied with the training contents aligning with your

expectations (pos-survey)?

21. Any further comments………………………….
